# Design of an In-Operando Cell for X-Ray and Neutron Imaging of Oxygen-Depolarized Cathodes in Chlor-Alkali Electrolysis

**DOI:** 10.3390/ma12081275

**Published:** 2019-04-18

**Authors:** Marcus Gebhard, Melanie Paulisch, André Hilger, David Franzen, Barbara Ellendorff, Thomas Turek, Ingo Manke, Christina Roth

**Affiliations:** 1 Institute of Chemistry and Biochemistry, Freie Universität Berlin, Takustraße 3, 14195 Berlin, Germany; christina.roth@fu-berlin.de; 2 Institute of Applied Materials, Helmholtz-Zentrum Berlin für Materialien und Energie GmbH, Hahn-Meitner-Platz 1, 14109 Berlin, Germany; melanie.paulisch@helmholtz-berlin.de (M.P.); hilger@helmholtz-berlin.de (A.H.); manke@helmholtz-berlin.de (I.M.); 3 Institute of Chemical and Electrochemical Process Engineering, Clausthal University of Technology, Leibnizstraße 17, 38678 Clausthal-Zellerfeld, Germany; franzen@icvt.tu-clausthal.de (D.F.); barbara.ellendorff@tu-clausthal.de (B.E.); turek@icvt.tu-clausthal.de (T.T.)

**Keywords:** in-operando and in-situ, X-ray and neutron imaging, cell design, chlor-alkali electrolysis, gas diffusion electrode, oxygen-depolarized cathode, oxygen reduction reaction

## Abstract

Oxygen-depolarized cathodes are a novel concept to be used in chlor-alkali electrolysis in order to generate significant energy savings. In these porous gas diffusion electrodes, hydrophilic and catalytically active microsized silver grains and a hydrophobic polytetrafluoroethylene cobweb structure are combined to obtain the optimum amount of three-phase boundaries between the highly alkaline electrolyte and the oxygen gas phase to achieve high current densities. However, the direct correlation between specific electrode structure and electrochemical performance is difficult. In this work, we report on the successful design and adaptation of an in-operando cell for X-ray (micro-computed tomography, synchrotron) and neutron imaging of an operating oxygen-depolarized cathode under realistic operation conditions, enabling the investigation of the electrolyte invasion into, and distribution inside, the porous electrode for the first time.

## 1. Introduction

Chlorine is a commodity chemical, needed in large quantities for the manufacture of more than 60% of all chemical products [1]. Until recently, it has been mainly synthesized by the amalgam and the diaphragm process [2,3]. These two versions of the chlor-alkali electrolysis (CAE) have been subject to development and research to minimize the harmful and controversial use of mercury and asbestos [4]. The membrane process developed in the 1970s [5] is environmentally friendly in regards to the used materials and more energy efficient than the older amalgam and diaphragm processes, but still huge amounts of electrical energy are required, resulting in high CO_2_ emissions [6]. In context to the efforts to reduce global greenhouse gas emissions, a novel approach has been proposed, by which the CO_2_ emissions can be reduced significantly [7]. Herein, an oxygen-depolarized cathode (ODC) replaces the hydrogen-evolving cathode in the CAE system. Electrical energy savings of up to 30% can be obtained due to the lower Gibbs free energy of the oxygen reduction reaction (ORR) compared to the hydrogen evolution reaction (HER). In this manner, the cell potential can be theoretically lowered by 1.23 V under standard conditions, resulting in savings up to 1 V under conditions relevant to industrial application, where the ODC is operated at 80 °C in 30 wt.% NaOH electrolyte. Considering the total energy demand for the chlor-alkali process, this technology could cut up to 1% of the total electrical energy demand in Germany [7,8]. The life cycle assessment of ODCs shows that the total energy and cost savings achieved will be an essential step forward in order to meet future emission goals [9,10,11]. 

The ODC is a special version of widely used gas diffusion electrodes (GDE) [12,13,14] with the oxygen reduction reaction taking place as the main reaction. Platinum is a commonly utilized, very efficient, and stable ORR catalyst, but for industrial CAE application a porous silver electrode has been proposed in the past because of its comparable activity and stability [15] in the highly alkaline electrolyte (30 wt.% NaOH). Even though silver is much less costly than platinum, a significant quantity of the catalytically active material is still required [16]. Consequently, an optimal catalyst utilization is of utmost importance. Since the catalyst utilization is directly linked to the number of particles, which are electrochemically addressed, and this is achieved by the distribution of three-phase boundaries, the porous electrode structure plays an important role. Consequently, it is essential to correlate the electrode’s 3D architecture with the electrochemical performance. In the ODC, the highly alkaline electrolyte and the gas phase get into contact at the three-phase boundary regions; ORR-active, electron-conducting, and hydrophilic µm-sized silver particles will form agglomerates that are partly filled with the electrolyte. The gas phase is introduced in the porous electrode by a hydrophobic polytetrafluoroethylene (PTFE) phase encompassing the silver particles in a cobweb-like fashion [16]. Routinely, such electrodes are prepared by airbrushing techniques, followed by a hot-pressing step to compact the structure and then a heat treatment procedure to obtain porous structures and to sinter the material. Since many parameters can be varied in the process, it is difficult to completely control the final electrode structure. Moreover, to our knowledge a direct and in-depth correlation between structure characteristics and electrochemical performance has not been made yet. 

Different imaging techniques have been used to characterize the 3D structure of porous electrodes (e.g. focused ion beam (FIB)/scanning electron microscopy (SEM) [17,18], X-ray as well as neutron tomography and radiography), each of them addressing a different length-scale with different resolution and specific advantages [19]. In particular, significant advances have been made in the area of fuel cells. Thus, it was possible to show, explain, and quantify the water transport within polymer electrolyte membrane fuel cells in-situ and in-operando with synchrotron X-ray [20,21,22,23,24] and neutron radiography [25,26,27,28,29,30,31,32]. However, the invasion and distribution of electrolyte within metallic GDEs and ODC in a realistic testing set-up have not yet been imaged during operation. Furthermore, due to the different phases, silver, and PTFE in the GDEs, it is necessary to ensure in-operando measurements with X-rays and neutrons. While X-ray measurements provide excellent spatial and time resolution [33], neutron radiography leads to a high contrast of the electrolyte to the metallic GDE, when NaOD is used as electrolyte and thus provides additional insight. To understand how ODCs work, it is of high importance to perform in-operando tests to image, analyze, and quantify the electrolyte flow within the GDE. 

In this paper, we report on the successful design and adaptation of an in-operando cell for X-ray and neutron imaging of an operating ODC under realistic operation conditions. We will describe the considerations based on which materials were chosen and how the cells were modified. The commercial Gaskatel cell served as a starting point and was optimized for the imaging process, leading to the first generation (1^st^ Gen.) cell. The further development to the second generation (2^nd^ Gen.) cell improved the electrochemistry features of the cell, so that the 2^nd^ Gen. shows an optimal compromise between the electrochemical and the imaging requirements.

## 2. Materials and Methods

### 2.1. Electrode Preparation

The ODCs investigated in this study were fabricated by an airbrushing process similar to [16]. In brief, an aqueous suspension of the microsized silver catalyst (SF9ED, Ferro, Frankfurt a. M., Germany) and PTFE (PTFE Dispersion TF 5060GZ, 3M Dyneon, Burgkirchen, Germany) as a hydrophobic agent were mixed with a methylcellulose solution (WALOCEL^®^ MKX 70000 PP 01, Dow Wolff Cellulosics GmbH, Bomlitz, Germany) as a pore building agent and thickener. The suspension was mixed with an Ultra-Turrax (IKA^®^-Werke GmbH & CO. KG, Staufen im Breisgau, Germany) and homogenized with sonication, then consecutively air-brushed onto a conductive supporting material (Ni-mesh 106 × 118 μm mesh size, 63 μm thickness, Haver & Boecker OHG, Oelde, Germany). The resulting electrodes were hot-pressed with a pressing load of 15 MPa at 130 °C for 5 min and sintered at 330 °C for 15 min to burn off the methylcellulose and improve the mechanical stability through PTFE sintering. The resulting ODC was subsequently cut into a rectangular shape (20 × 20 mm, 350 µm thickness) to fit into the half-cell set-up.

### 2.2. Electrochemical Characterization

Electrochemical characterization was performed in a three-electrode set-up using either the commercial GDE testing cell (FlexCell^®^ HZ-PP03, Gaskatel GmbH, Kassel, Germany) or the newly developed in-operando cells (1^st^ Gen. cell and 2^nd^ Gen. cell). A potentiostat (Gamry Ref 3000, Gamry Instruments, Warminster, PA, USA) was used for electrochemical measurements. As the working electrode (WE), silver ODCs as mentioned above, with a geometric surface area of 3.14 cm² were utilized. A platinum wire electrode (99.95% purity, 1 mm diameter, 25 cm length) was used as a counter electrode (CE) and a HydroFlex^®^ (Gaskatel GmbH, Kassel, Germany) reversible hydrogen electrode (RHE) represented the reference electrode (RE). The industrially used electrolyte (30 wt.% NaOH solution) was prepared by dissolution of caustic flakes (> 99% NaOH, Carl Roth, Germany) in deionized water (Merck Millipore, Burlington, MA, USA) and provided to the electrolyte side of the ODC. On the backside of the cathode, oxygen (≥99.5%; Air Liquide, Paris, France) was provided via a pressure controller (Everwand Druckgastechnik GmbH, Solingen, Germany) by constant flow of 20–50 mL min^−1^ (Influx Measurements Ltd., Alresford, UK) and minimal backpressure using a 5 mm water column. 

For electrode characterization the following electrochemical methods were applied, as single procedure or periodically scripted procedures for long-term measurements. The operating temperature of the cell was set to 20, 50, or up to 80 °C by regulation of cell and electrolyte tank temperature. Linear sweep voltammetry (LSV) measurements were performed either without correction for inner resistance (iR) or in current interrupt (CI) mode. Starting at open circuit (1.0–1.1 V vs. RHE), the potential was changed with a constant sweep rate of 1 or 2 mV s^−1^ to a lower limit of approximately 0.2 V vs. RHE. Depending on the iR correction, or current limitation of the potentiostat, the measurements were terminated before reaching the lower potential limit. Long-term operation chronoamperometric measurements (CA) were performed at different potentials (0.2, 0.5, and 0.9 V vs. RHE, not iR corrected) up to 1 h per potential. For post iR compensation of the electrochemical measurements, potentiostatic electrochemical impedance spectroscopy (EIS) was performed in a range from 1 MHz to 100 mHz with an AC voltage of 5 mV at 0.2–0.9 V vs. RHE respectively. 

### 2.3. Micro-CT Measurements

**Figure 1:**
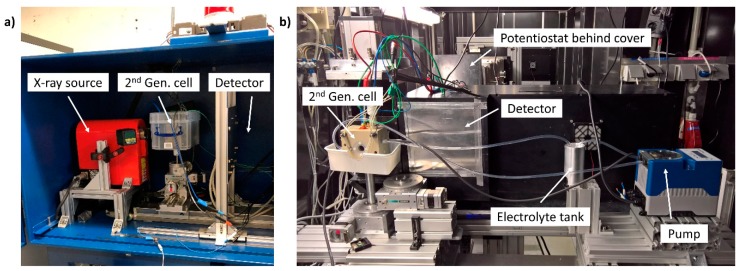
Photographs of the experimental in-operando set-up (**a**) micro-computed tomography at Helmholtz-Zentrum Berlin; (**b**) neutron radiography at CONRAD2 Berlin.

The laboratory X-ray micro-computed tomography (micro-CT) radiography set-up consisted of a Hamamatsu X-ray tube (L8121-03, tungsten anode, Hamamatsu Photonics K.K., Hamamatsu, Japan) and a Hamamatsu flat panel detector (C7942SK-05, Hamamatsu Photonics K.K., Hamamatsu, Japan). The in-operando cell was placed between the source and detector as seen in Figure 1a. The half-cell was furthermore shielded by a modified plastic housing, providing sufficient protection of the measurement environment for possible electrolyte contamination. The separate electrolyte tank (PTFE container, 250 mL) was placed in a heating bath outside the micro-CT housing, while the peristaltic pump, oxygen flow controller, temperature controller unit and the potentiostat were connected and placed in the micro-CT housing. The magnification achieved in this way was the largest that projected the entire field of view onto the detector, resulting in a pixel size of 12.25 µm. To achieve maximum contrast and the best signal-to-noise ratio, the X-ray tube was operated at 130 kV and at 230 µA with a 0.5 mm copper filter. The normalization of the captured images was performed using the imaging software Fiji (v1.5.2) [34]. 

### 2.4. Synchrotron Radiography

Preliminary synchrotron experiments without operating the ODC were conducted at the BESSYII BAMLine to demonstrate suitable resolution and the experimental feasibility. To simulate the electrolyte behavior in these preliminary experiments, the 1^st^ Gen. cell was flooded with deionized water. For the experiments, a monochromatic photon energy of 25 keV was used. The optical set-up, consisting of a (4008 × 2672 pixel) CCD camera (PCO 4000, PCO AG, Kelheim, Germany) and a cadmium tungstate (CdWO_4_) scintillator screen were used to capture images with a pixel size of 985 nm. Resulting images were calculated using the imaging software Fiji (v1.5.2) [34].

### 2.5. Neutron Radiography

Neutron experiments were carried out at the CONRAD/V7 Beamline facility of the Helmholtz-Zentrum Berlin, Institute of Applied Materials (BER II research reactor). The in-operando cell was operated at 20 °C. The half-cell was connected to the oxygen and electrolyte supply (30 wt.% NaOD in D_2_O, 99 at.% D, Sigma Aldrich, St. Louis, MI, USA), as well as the potentiostat. A specially prepared shielding, consisting of a modified thin plastic housing was provided and connected to the sample holder to secure the measurement environment for possible electrolyte contaminations (see Figure 1b). Images of the ODC were taken with white beam at 2 to 6 Å (peak at approximately 3.5 Å). The neutrons were detected at a gadolinium oxide (Gd_2_O_3_) coated scintillator in combination with a Neo 5.5 sCMOS camera (Andor, Oxford Instruments, Abingdon, UK) with 5.5 megapixel providing a pixel size of 6.5 µm. 

## 3. Results and Discussion

### 3.1. General Considerations

For characterization and imaging of all processes taking place at an ODC, a rather complex set up is required. In CAE where ODCs are utilized, the appropriate supply of gaseous oxygen to one side of the ODC and liquid electrolyte to the other side are critical operating factors. Furthermore, the cell set-up needs to be stable against corrosion caused by the highly alkaline electrolyte (30 wt.% NaOH) at temperatures up to 80 °C [11,16]. In addition, a sophisticated periphery is necessary to keep the process conditions constant for electrochemical characterization and during imaging of the temperature sensitive process. Consequently, the reliable characterization of the electrochemical performance of ODCs for ORR can only be achieved if the half-cell set-up is constructed in a way to guarantee constant measurement conditions. Additionally, a three-electrode set-up has to be used to ensure the accurate measurement of the ODC characteristics. Therefore, the set-up has to include the working electrode (ODC), a reference electrode compatible to the operating conditions, and a suitable and stable counter electrode. The positioning of the electrodes should be done carefully to minimize their influence on the electrochemical measurement (i.e., not causing a large ohmic drop and disturbance of the electrical field).

The cell geometry and position of all components play an important role when imaging the physical processes taking place at the ODC. In general, the set-up has to be constructed in a way that the incoming beam of either X-rays or neutrons is absorbed in a minimal way. The absorption of the incident beam can be caused by the cell material, cell components, the electrolyte, or the ODC itself. For radiography investigation with X-ray radiation, in-plane measurements were excluded, due to the strong absorption of the electrode material (in-plane 2 cm of silver and nickel). If radiography imaging is used to investigate the electrolyte behavior in through-plane geometry, the ODC structure exerts less impact (electrode thickness 350 µm). Therefore, the experiment has been designed in a way that the beam direction is perpendicular to the electrode with a minimized impairment by components in beam direction.

If all of these requirements regarding the operating conditions, electrochemical measurements and the imaging are met, an investigation of the electrolyte behavior during the operation of an ODC at technically relevant conditions is possible. 

### 3.2. Gaskatel Cell

**Figure 2:**
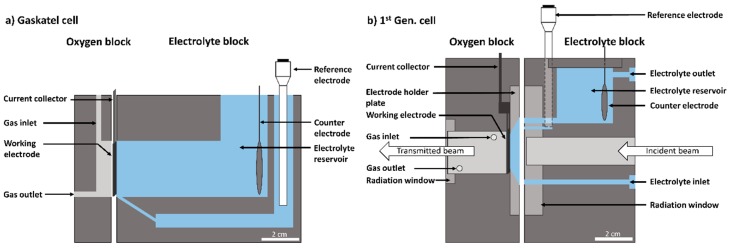
Schematic diagram of (**a**) Gaskatel cell and (**b**) first generation (1^st^ Gen.) cell with relevant parts.

A well-established half-cell set-up for the electrochemical characterization of GDEs is provided by Gaskatel. The commercially available half-cell (FlexCell^®^) allows to measure under industrially relevant conditions (30 wt.% NaOH, 80 °C) with a three-electrode set-up in a chemically resistant cell environment. The cell consists of two compartments, one for the supply of oxygen to the ODC and an electrolyte compartment on the opposite side of the ODC for the electrolyte supply (see Figure 2a). However, the reference electrode, counter electrode, electrolyte reservoir, and other parts of the Gaskatel cell are placed in positions, where the beam would have to pass for through-plane radiography imaging (see Figure 2a). Therefore, the cell set-up has to be modified significantly to allow imaging of the electrode without constraints due to the cell components.

### 3.3. Development of 1^st^ Gen. Cell

The position of key components in the commercial Gaskatel cell does not allow for the radiography imaging of operating ODCs. The focus for developing the 1^st^ Gen. cell (see Figure 2b) was on adapting the existing design in a way that it attenuates the radiation as little as possible, while keeping the effect on the electrochemical measurement as low as possible. Therefore, the novel design was divided into two compartments, like the Gaskatel cell, to keep its functionality; but with the electrolyte, CE and RE were positioned differently to allow for successful imaging. 

Polyether ether ketone (PEEK) was chosen as the material for the half-cell, because of its high resistivity against alkaline solutions, good thermal stability, and good machinability. PEEK also provides good durability under X-ray radiation [35] and was chosen as material for components remaining in the path of the beam. The strongly absorbing electrolyte reservoir with the counter electrode was placed above the beam channel to minimize the electrolyte volume in the beam path. However, a thin layer of electrolyte in front of the electrode could not be completely eliminated, as it is required for the ODC to operate properly. Consequently, a conically-shaped electrolyte chamber with 5 mm thickness was placed in front of the ODC. Due to the small volume, the electrolyte had to be pumped to maintain stable measurement conditions. The electrolyte inlet was positioned below the beam with the electrolyte flowing onto the electrolyte holder plate, not directly onto the ODC surface. This has another beneficial side effect that gas bubbles passing through the ODC will collect at the top of the electrolyte chamber and will be pumped in the flow direction with evolving gas from the CE into the outlet. In addition, the reference electrode was positioned beside the electrolyte reservoir and connected to the electrolyte chamber in front of the ODC by a capillary. In this way, important components could be eliminated from the beam path, while the three-electrode set-up remained operational. As a result, the incident beam can pass the electrolyte block with minimal attenuation. The radiation window of the electrolyte block is milled to a minimum thickness of 1 mm, which the beam has to pass, before entering the 5 mm thick electrolyte chamber and the ODC. Behind the ODC, the gas supply compartment is situated and designed to tighten the top of the electrode mesh with the current collector and to keep the ODC in position with a screwed holder plate. Gaskets between the block and holder plate seal the ODC surface and restrict it to 3.14 cm². The working electrode area is the same as in the Gaskatel cell and was not changed to obtain a wider field of view on the electrode surface. After interaction of the beam with the ODC, the beam exits at the backside of the ODC to a cylindrical oxygen reservoir with connections to the gas supply. A sealed radiation window, milled in the direction of the beam to a thickness of 1 mm and therefore, absorbing as little of the beam as possible, closes the reservoir. From Figure 2b it can be seen that the incident beam passes only 2 mm of block material, the 5 mm thick electrolyte chamber in front of the ODC, and the ODC itself. In this way, the absorption of the incident beam by the half-cell was kept to a minimum, resulting in promising results obtained from preliminary X-ray radiography measurements.

However, not included in the schematic figures, but important for the operation of the cell, are seals, heating cartridges, and tubing connections, as well as the temperature sensors. Heating cartridges were placed in the electrolyte block near the electrolyte reservoir and inlet to allow heating up to 80 °C, regulated by a separate temperature controller. In this way, the developed set-up was optimized for radiography imaging and met all requirements for operating the cell at technically relevant electrochemical conditions.

### 3.4. Preliminary Synchrotron Measurements

**Figure 3:**
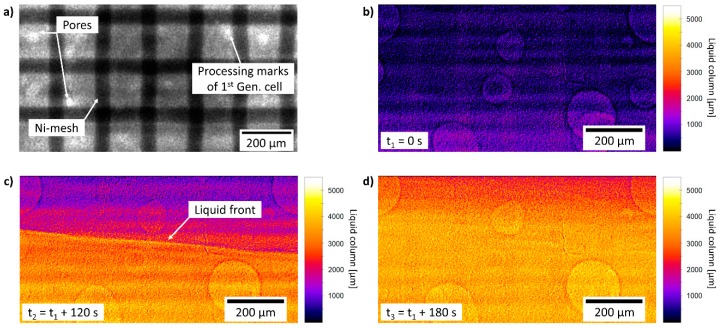
Images of oxygen-depolarized cathode (ODC) obtained by synchrotron radiation in 1^st^ Gen. cell, (**a**) microstructure of ODC as obtained; (**b**–**d**) normalized (by dry cell) images of filling process of 1^st^ Gen. cell with deionized water, the liquid front is clearly visible.

The 1^st^ Gen. cell was investigated at the synchrotron BESSYII to test the achievable resolution and the experimental feasibility. Figure 3a presents an image of the GDE with its microstructure clearly visible. The nickel mesh (which is utilized as conducting and mechanically supporting structure) appears clearly, also some artifacts due to the manufacturing of the radiation windows. In particular, some parts of the radiation windows are not fully planar, which is caused by the milling process. However, this in-situ measurement demonstrated the suitability of our cell concept. Additionally, in Figure 3b–d the cell is flooded with deionized water to demonstrate that the electrolyte shows enough contrast, so that its interaction with the ODC can be imaged. Already in this experiment, different structural features of the ODC can be observed. For example, relatively large pores appear in between the mesh structure of the Ni-substrate.

### 3.5. Electrochemical Testing of 1^st^ Gen. Cell

While the 1^st^ Gen. cell turned out to be a suitable set-up concerning material and imaging properties, our next aim was to apply in-operando imaging during the operation of the ODC at industrially relevant conditions. For operation and electrochemical characterization of ODCs different electrochemical methods, such as LSV and CA, can be applied. LSVs reveal the performance of the ODC for the ORR. Each measurement is affected by the iR of the set-up, reducing the effective potential that is applied to the ODC. Therefore, the LSVs have to be corrected in order to compare the performance of electrodes in different set-ups. Commonly, the measurements are either post-corrected by a separately determined iR value or corrected by the operating program of the potentiostat (CI-mode) directly during the measurement. For post-correction, the iR can be evaluated by additional EIS measurements and be used for correction of the measured LSVs in the analysis program. In contrast, the CI-mode interrupts the LSV measurement for a short time and compensates the LSV instantaneously for the determined iR-value. 

**Figure 4:**
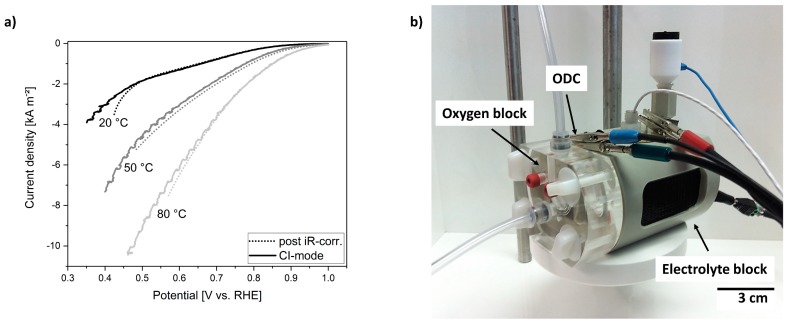
(**a**) Linear sweep voltammograms (LSV) of technical ODC in Gaskatel cell (20, 50, and 80 °C, 30 wt.% NaOH, correction of applied potential by post-correction (dotted lines) or CI-mode (full lines), sweep rate 2 mV s^−1^); (**b**) photography of operating Gaskatel cell.

For comparison of the different cell set-ups, ODCs were characterized in the Gaskatel cell and 1^st^ Gen. cell by LSVs under the same conditions (20, 50, and 80 °C, 30 wt.% NaOH). The correction of the potential is either done by post-correction for one determined iR value or measured in CI-mode. LSVs obtained with the Gaskatel cell of a technical ODC are shown in Figure 4a. The results reveal distinct differences between the two iR correction modes. During characterization, ODCs achieve high current densities and as a result of the current flow and the iR, the electrolyte heats up. Thus, the iR value of the set-up changes during the measurement to a lower resistance. Consequently, the post-correction leads to erroneous results, as the LSV curves are corrected for one false iR value (dotted lines). However, the CI-mode, designed to avoid this problem, faces another issue. The electrolyte in the Gaskatel cell (see Figure 4b) is not pumped, but remains stationary so that evolving oxygen from the counter electrode will stay inside the electrolyte and hinder the correction for the right iR value. The determination of the iR is disturbed by the oxygen bubbles and as a result the potentiostat corrects the LSVs for changing and false iR values, leading to noisy measurements (full lines). Hence, it can be shown that the characterization of ODCs using the Gaskatel cell is problematic at high current densities above 5 kA m^−^². The heating up of the electrode and electrolyte during operation and the evolving gas bubbles are a big disadvantage of the Gaskatel cell, when measurements at high current densities are required. 

Even though heating up of the electrolyte was remedied by pumping electrolyte through the cell, and evolving gas on the counter electrode was remedied by purging of the electrolyte reservoir in the 1^st^ Gen. cell design, electrochemical testing with an ODC at technical current densities revealed that the flow area chosen between the working and counter electrodes was still too small in scale. As a result, the cell resistance between the working and counter electrodes was high, limiting the operation of the ODC to current densities below 4 kA m-². Pumping the electrolyte could not compensate for the small exchange area between the working and counter electrode, making measurements at high current densities impossible with the chosen potentiostat. These results show that operation of ODCs at high current densities is highly problematic, leading to the 2^nd^ Gen. cell with further improved design features. 

### 3.6. Development of 2^nd^ Gen. Cell

**Figure 5:**
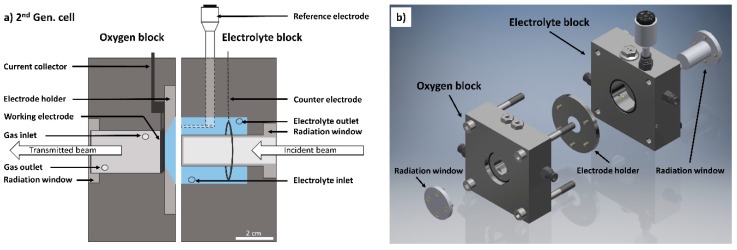
(**a**) Schematic diagram of second generation (2^nd^ Gen.) cell with relevant parts; (**b**) 3D-cell design of 2^nd^ Gen. cell.

To enable the operation and electrochemical characterization of ODCs at temperatures up to 80 °C and 30 wt.% NaOH, the design of the 1^st^ Gen. cell was revised (Figure 5a). We focused on increasing the exchange area between the working and counter electrode to allow for measurements at high current densities. The suggested new design is shown in 3D in Figure 5b. The position of the electrolyte reservoir and counter electrode, as well as the shape of the radiation window, were modified. The reservoir was moved into the center of the block, surrounding the radiation window. The existing conical electrolyte chamber was extended by a tubular reservoir providing an increased exchange area between WE and CE. The ring-shaped counter electrode around the radiation window provides a more homogeneous electrical field, which is favorable for the electrochemical measurement. The pump inlet and outlet are placed perpendicular to the direction of the beam. The top position of the outlet allows for the removal of evolving gas from the CE. The components of the cell were modified as outlined, but the material in the direction of the beam (2 mm radiation window material and 5 mm electrolyte volume) was kept identical to the 1^st^ Gen. cell design. The imaging properties of the cell should; therefore, remain the same.

**Figure 6:**
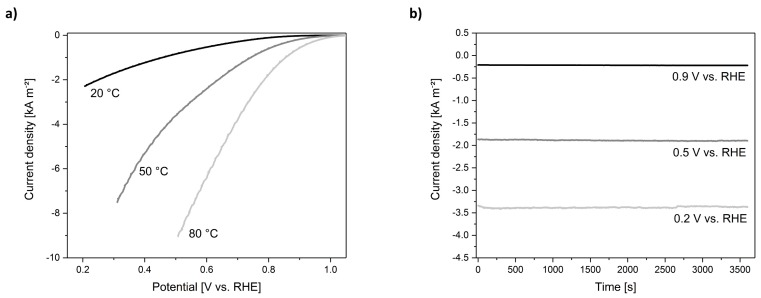
Electrochemical characterization in 2^nd^ Gen. cell (**a**) LSV of ODC recorded up to high current densities (20, 50, and 80 °C, 30 wt.% NaOH, correction of applied potential by CI-mode, sweep rate 2 mV s^−1^); (**b**) Chronoamperometry (CA) at 0.2, 0.5, and 0.9 V vs. RHE (80 °C, 30 wt.% NaOH, no iR-correction) showing very stable features.

Electrochemical investigation using the newly designed cell showed significant improvements with respect to the 1^st^ Gen. cell. The cell set-up could be used at every temperature without showing any corrosion effects. Furthermore, the modified cell design demonstrates a significant improvement when measuring LSVs up to high current densities. In Figure 6a, the LSVs of technical ODC are compared for three different operating temperatures. The ORR activity increased with temperature, reaching current densities up to 9 kA m^−^². The LSVs were recorded using the CI-mode. Contrarily to 1^st^ Gen. cell, we did not observe any drawbacks, such as a limited current density, due to the cell design. To demonstrate that the cell can be used reliably to perform long-term measurements while applying a constant potential, CA measurements were carried out at different temperatures. In Figure 6b it is shown that at 80 °C and three different applied potentials, stable measurements can be achieved for several hours. The applied potentials in the CA measurements were not corrected for iR, but were chosen to represent different operation conditions. In brief, the 2^nd^ Gen. cell set-up fulfills all requirements for the detailed and systematic imaging of processes occurring during ODC operation in technically relevant conditions. 

### 3.7. Micro-CT testing of 2^nd^ Gen. Cell

**Figure 7:**
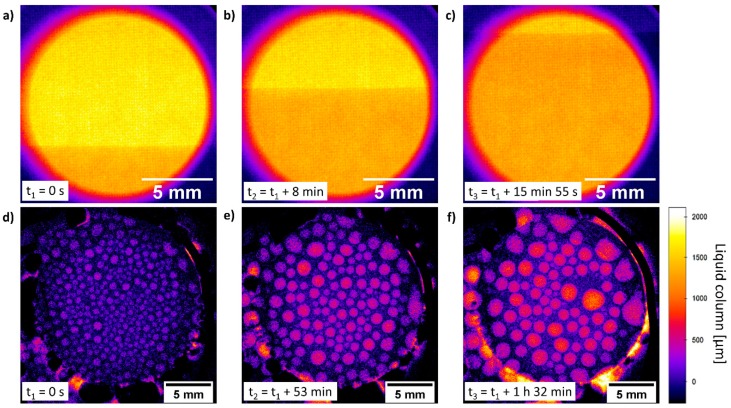
(**a**–**c**) Flooding process of the 2^nd^ Gen. cell, recorded using a lab-scale micro-CT. (technical ODC, 30 wt.% NaOH solution); (**d**–**f**) in-operando radiography measurements at 75 °C.

The cell set-up was further tested while performing radiographies in a lab-scale micro-CT. In Figure 7a–c the flooding of the in-operando 2^nd^ Gen. cell with 30 wt.% NaOH electrolyte is displayed. For this purpose, a technical ODC was mounted in the 2^nd^ Gen. cell and the electrolyte was pumped at a reduced pumping rate to obtain a higher time resolution. The flooding process within the field of view took 23 min using an exposure time of 2.5 s per frame. The ODC structure is visible and the fine mesh structure of the conducting Ni-substrate appears to be regular. The rising electrolyte front is recognizable from the higher absorption of the X-ray by the electrolyte and represented by a darker color. The in-operando cell was operated with different technical electrodes and operating conditions for several days without any problems. During this process, images of the operating ODCs were taken (Figure 7d–f) at different time stages. These results represent the interaction of the electrode structure with the electrolyte. The appearing droplets could be formed by evaporation and condensation of electrolyte on the colder backside of the electrode. A more detailed investigation and discussion will be subject of a forthcoming publication by Paulisch et al. 

### 3.8. Neutron Testing

**Figure 8:**
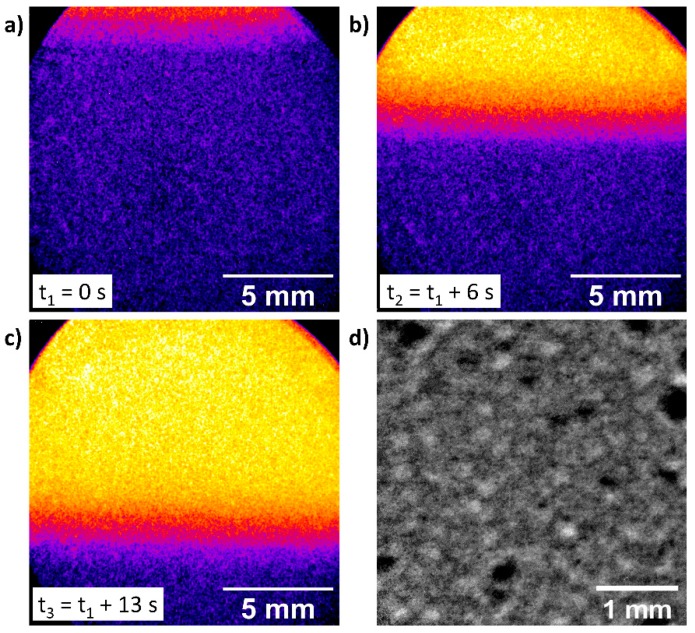
(**a**–**c**) Neutron radiography of pumping process in 2^nd^ Gen. cell; (**d**) in-operando neutron radiography image of ODC.

In addition to the radiography measurements with X-rays, the cell should also enable imaging using neutrons, as complementary information can be obtained. However, testing the cell with neutron radiation is only possible with a modified cell set-up, adapted to the respective requirements. Since neutron radiation is strongly absorbed by hydrogen- and carbon-containing materials, the cell materials had to be replaced, in parts, with more suitable polymers. PEEK consists of a significant amount of carbon and hydrogen in its monomer structure. Thus, it had to be exchanged. A possible alternative material is polychlorotrifluoroethylene (PCTFE), since it provides a sufficient resistivity to the highly alkaline electrolyte (up to 80 °C) and a high material hardness, making it suitable to be processed. Additionally, its low carbon content and absence of hydrogen in the polymer will result in a low absorption of the neutron beam. The standard electrolyte, an aqueous solution of 30 wt.% NaOH, would absorb a large fraction of the beam. Therefore, the in-operando tests with neutron radiation have been performed using an electrolyte of 30 wt.% NaOD in D_2_O. With the replacement of the electrolyte, the in-operando set up is also suitable for neutron imaging and was tested at CONRAD2 beamline (see experimental set-up Figure 1b). In Figure 8a–c the process of emptying the 2^nd^ Gen. cell from deuterated electrolyte is demonstrated and shows the suitability of the cell for neutron radiography. A clearly visible electrolyte contrast and interaction of electrolyte with the ODC structure can be demonstrated and detected. Furthermore, in Figure 8d an inverted in-operando radiography image is presented to show the detectable electrolyte contrast. Bright spots indicate a higher amount of absorbing electrolyte in the beam path. 

In Table 1, capabilities of each previously described cell design and set-up are summarized. The advantages of the 1^st^ Gen. cell, over the commercial Gaskatel design, were the changes to the electrolyte and gas supply. By careful design, it was possible to have the lowest amount of absorbing material in the beam path. Most requirements for successful imaging were being met, and the 1^st^ Gen. cell enabled in-situ radiography imaging of ODC for the first time. However, it was not possible to meet the necessary electrochemical requirements, in particular high current densities. The further improvement to 2^nd^ Gen. cell allowed in-operando imaging with both X-ray and neutron radiation at industrially relevant conditions for the first time. Therefore, the 2^nd^ Gen. cell enabled us to investigate the electrolyte invasion and flooding taking place during the operation of an ODC.

## 4. Conclusions and Outlook

In-operando cells could be devised and optimized to allow for the collection of X-ray and neutron imaging data during operation of ODCs in highly alkaline conditions. While the 1^st^ Gen. cell still suffered from a high cell resistance, in a second approach, the electrolyte reservoir was modified to allow for the beam to pass unhindered through the cell and the cell performance was significantly improved. The 2^nd^ Gen. cell demonstrated reliable, stable and reproducible LSV curves in the X-ray imaging sample environment. For allowing neutron imaging, the beam windows were manufactured in a fashion that they can be easily exchanged to less neutron-absorbing polymer types. First X-ray and neutron imaging results of an operating ODC in realistic working conditions were obtained and demonstrated the filling of the porous electrode structure with the liquid electrolyte. In future experiments we will focus on the investigation of the electrolyte behavior during electrochemical measurements, applying different electrode structures and compare pristine and aged electrodes.

## Figures and Tables

**Table 1:** Development of cell capabilities.

Cell Requirements	Gaskatel Cell	1^st^ Gen. Cell	2^nd^ Gen. Cell
Resistivity to alkaline media	✓	✓	✓
Electrolyte and gas supply	(✓)	✓	✓
Electrochemical characterization	(✓)	(✓)	✓
Imaging radiation:			
X-ray (PEEK windows)	✗	(✓)	✓
Neutron (PCTFE windows)	✗	(✓)	✓

✓ Suitable, (✓) suitable with restrictions, ✗ not suitable.
